# Neural Regeneration in Dry Eye Secondary to Systemic Lupus Erythematosus Is Also Disrupted like in Rheumatoid Arthritis, but in a Progressive Fashion

**DOI:** 10.3390/ijms241310680

**Published:** 2023-06-26

**Authors:** Balázs Sonkodi, László Marsovszky, Anita Csorba, Attila Balog, Bence Kopper, Zoltán Zsolt Nagy, Miklós D. Resch

**Affiliations:** 1 Department of Health Sciences and Sport Medicine, Hungarian University of Sports Science, 1123 Budapest, Hungary; 2 Department of Ophthalmology, Semmelweis University, 1085 Budapest, Hungary; 3 Department of Rheumatology and Immunology, Faculty of Medicine, Albert Szent-Györgyi Health Center, University of Szeged, 6725 Szeged, Hungary; 4 Faculty of Kinesiology, Hungarian University of Sports Science, 1123 Budapest, Hungary

**Keywords:** dry eye disease, systemic lupus erythematosus, autoimmune disease, Langerhans cell, Piezo2, Piezo1, channelopathy

## Abstract

Our objective in this study was to analyze the aberrant neural regeneration activity in the cornea by means of in vivo confocal microscopy in systemic lupus erythematosus patients with concurrent dry eye disease. We examined 29 systemic lupus erythematosus patients and 29 age-matched healthy control subjects. Corneal nerve fiber density (CNFD, the number of fibers/mm^2^) and peripheral Langerhans cell morphology were lower (*p* < 0.05) in systemic lupus erythematosus patients compared to the control group. Interestingly, corneal nerve branch density, corneal nerve fiber length, corneal nerve fiber total branch density, and corneal nerve fiber area showed a negative correlation with disease duration. A negative correlation was also demonstrated between average corneal nerve fiber density and central Langerhans cell density. This is in line with our hypothesis that corneal somatosensory terminal Piezo2 channelopathy-induced impaired Piezo2–Piezo1 crosstalk not only disrupts regeneration and keeps transcription activated, but could lead to Piezo1 downregulation and cell activation on Langerhans cells when we consider a chronic path. Hence, Piezo2 containing mechanosensory corneal nerves and dendritic Langerhans cells could also be regarded as central players in shaping the ocular surface neuroimmune homeostasis through the Piezo system. Moreover, lost autoimmune neuroinflammation compensation, lost phagocytic self-eating capacity, and lost transcription regulation, not to mention autoantibodies against vascular heparin sulfate proteoglycans and phospholipids, could all contribute to the progressive fashion of dry eye disease in systemic lupus erythematosus.

## 1. Introduction

Systemic lupus erythematosus (SLE) is a multisystemic autoimmune disease with incredibly varied clinical manifestations and a diverse pathogenesis. The disease is characterized by the loss of self-tolerance and formation of nuclear and cytoplasmic autoantigens as well as immune complexes resulting in inflammation of multiple organs. The etiology of SLE is yet to be elucidated, but we know that genetic, hormonal, and environmental factors may play a key role [1]. Both innate and adaptive immune systems are involved in the early phase leading to the activation of T and B cells, which become self-sustained by a chronic self-aimed immune response resulting in the overproduction of proinflammatory cytokines [2]. Dry eye disease (DED), another debilitating disease, is also characterized by the uncontrolled overproduction of proinflammatory cytokines on the ocular surface, causing various degrees of distress to patients [3]. Wang et al. demonstrated in their meta-analysis of twenty-nine SLE studies that nearly a quarter of patients with SLE reported dry eye symptoms or presented abnormal Schirmer’s test findings, and that is higher than in the general population [4].

It is known from previous studies that being part of the lacrimal functional unit, corneal subbasal nerves have a pivotal role in the regulation of corneal sensation, maintenance of epithelial integrity, proliferation, and the promotion of wound healing [5]. Further to these, recent studies postulate their potential sensory afferent role in immunoregulatory processes via neuropeptides, such as vasoactive intestinal peptide (VIP) and pituitary adenylate cyclase-activating polypeptide (PACAP), that are typically released from nerve terminals [6,7]. Indeed, spinal sensory afferent neurons are known to have efferent and trophic functions, too [8]. The neural control of ocular immune privilege was initially proposed by Streilein et al. [9], and the evidence is strong that these peptides can exhibit anti-inflammatory properties by controlling proinflammatory cytokine production by leukocytes and other resident cells of the ocular surface.

One initial critical mechanism that may be involved in the immunoregulatory processes is sensory afferent mechanotransduction. The groundbreaking work of Patapoutian and his team attracted great attention among researchers with the discovery of Piezo ion channels [10], not to mention that they also identified the Piezo2 ion channel as a principal ion channel responsible for proprioception [11]. Piezo2 has a central role in sensory processes, and its theorized excessive mechanotransduction-derived non-contact microinjury could lead to a wide array of diseases, including even autoimmune conditions [12,13]. It has been postulated that the channelopathy of Piezo2 at somatosensory terminals contributing to proprioception may initially be responsible for the cascade of events that could lead to the activation of the innate [14] and adaptive immune system and eventually to uncontrolled production of autoantigens [15]. However, it is important to note that earlier, it was postulated that DED could also be induced as a result of peripheral Piezo1 channelopathy leading to neural Piezo2 channelopathy, hence constructing the base of the spectrum nature of DED [16].

Furthermore, Piezo2 containing somatosensory nerves have been proposed to carry special genetic identification [17,18] and suggested to be principal transcription activators in proprioceptive terminals in order to support remodeling and regeneration [19]. However, chronic Piezo2 channelopathy not only keeps transcription activated and wound healing unfinished, but is also postulated to disrupt the crosstalk with Piezo1 of peripheral cells [16]. As a result, Piezo1 of keratinocytes could be upregulated [13], as could be the case with corneal keratinocytes as well [15]. On the contrary, Piezo1 of dendritic cells (DC) is downregulated [20], as could be the case in corneal dendritic Langerhans cells (LC) [15]. If genetic or environmental factors, like repetitive re-injury, even non-contact ones, interferes with the due closure of this process, then remodeling and regeneration could be altered and/or disrupted [19], as is observed in DED secondary to rheumatoid arthritis (RA) [15]. Another proposed consequence of this lost Piezo2–Piezo1 crosstalk is the osmotic pressure changes on the ocular surface secondary to DED [16,21].

The role of corneal neuroimmune interactions in the development of DED is beyond doubt; however, we know little about the exact mechanisms that are operational in autoimmune diseases like SLE. Chronic Piezo2 channelopathy on corneal somatosensory terminals may be the missing puzzle that can lead to minute changes in the neuroimmune communicating network of the cornea, especially in autoimmune conditions like SLE. Therefore, our aim was to explore subtle morphological changes to this network by means of in vivo corneal confocal microscopy.

## 2. Results

The demographics and clinical data of patients are summarized in Table 1. No significant difference was found in reference to age distribution. All dry-eye parameters indicated DED; the Schirmer test results and the tear break-up time (TBUT) were lower, while the ocular-surface disease index (OSDI) score and lid-parallel conjunctival folds (LIPCOF) showed a higher value in the SLE group compared to the controls.

### 2.1. Comparison of SLE and Control Groups

**Figure 1:**
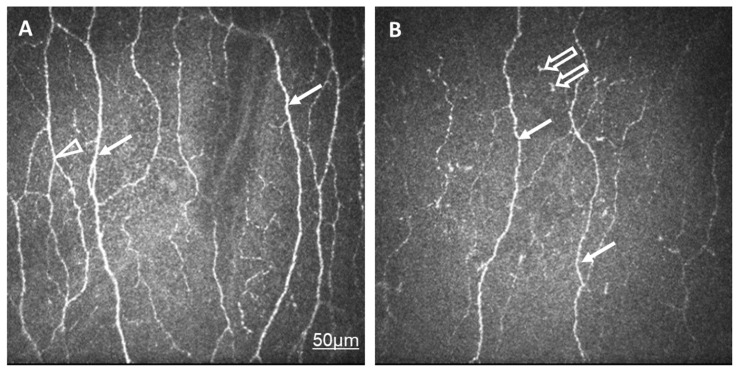
In vivo confocal corneal microscopic records in a healthy control eye (**A**) and in an eye of a patient with SLE and dry eye (**B**). The density and thickness of subepithelial nerve plexi (arrow) and the number of branches (arrowhead) are reduced in SLE compared to normal eyes. Langerhans cells are present in SLE (empty arrow). The same scale bar is applicable to Figure 1A,B.

Corneal nerve fiber density (CNFD, the number of fibers/mm^2^) and peripheral LC morphology were lower in the SLE group compared to the control group. Other variables, such as corneal nerve branch density (CNBD, the number of branch points on the main fibers/mm^2^), corneal nerve fiber length (CNFL, the total length of the nerves in mm/mm^2^), corneal nerve fiber total branch density (CTBD, the total number of branch points/mm^2^), corneal nerve fiber area (CNFA, the total nerve fiber area in mm^2^ per mm^2^) did not show any significant difference (Table 2, Figure 1).

**Table 2:** Mean and SD values for the confocal microscopic findings of the SLE and control samples, with *p* values of the comparisons (Mann–Whitney test). CNFD: the number of fibers per mm^2^; CNBD: the number of branch points on the main fibers per mm^2^; CNFL: the total length of nerves in mm per mm^2^; CTBD: the number of branch points per mm^2^; and CNFA: the total nerve fiber area in mm^2^ per mm^2^. LCD: Langerhans cell density. LC Morph: LC morphology. * *p* < 0.05 Mann–Whitney test.

	Control	SLE	*p*
CNFD	19.8 ± 8.3	15.3 ± 6.8	0.038 *
CNBD	26.7 ± 18.4	22.4 ± 17.3	0.294
CNFL	13.5 ± 4.1	11.5 ± 4.1	0.140
CTBD	44.1 ± 25.7	41.3 ± 33.7	0.423
CNFA	0.006 ± 0.002	0.005 ± 0.002	0.173
Central LCD	37.9 ± 45.4	32.9 ± 41.9	0.846
Peripheral LCD	95.7 ± 76.6	92.1 ± 56.6	0.768
Central LCM	1.0 ± 0.7	1.1 ± 0.5	0.611
Peripheral LCM	2.2 ± 0.5	1.4 ± 0.6	0.001 *

### 2.2. Subgroup Analysis by Disease Activity (SLEDAI Score)

Two subgroups were created according to the disease activity. If the SLEDAI score was 0, the disease was considered inactive; if it was >0, then the disease was considered active. No significant difference was found either in the corneal nerve parameters or in the LC parameters (Table 3).

### 2.3. Correlation Analysis

**Figure 2:**
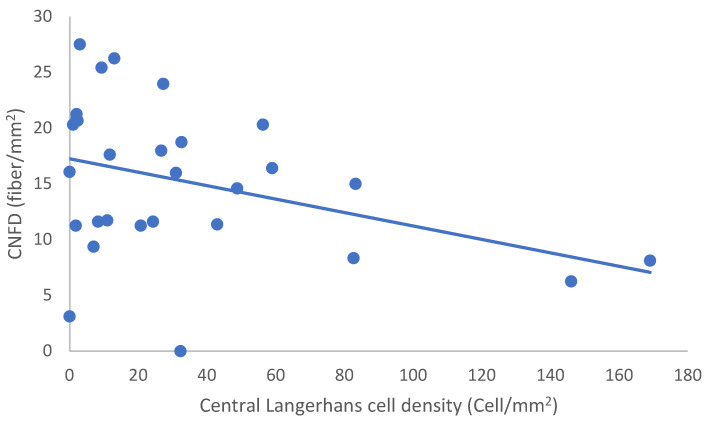
Correlation of CNFD and central LCD.

**Figure 3:**
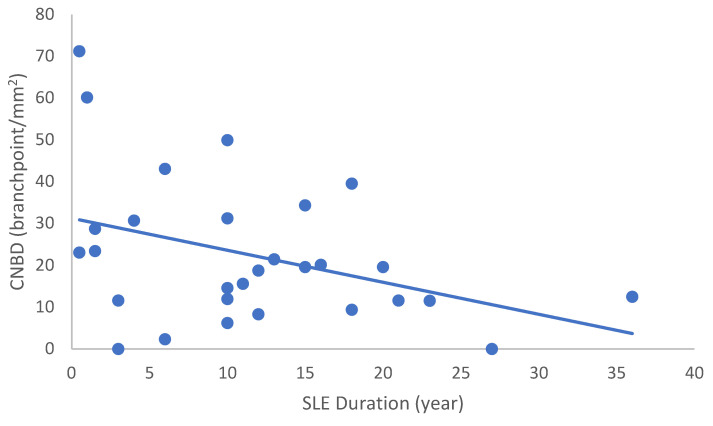
Correlation of CNBD with disease duration (r = −0.389; *p* = 0.040).

**Figure 4:**
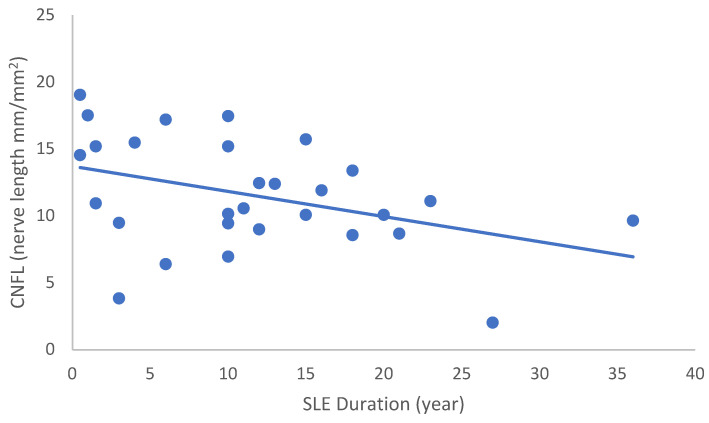
Correlation of CNFL with disease duration (r = −0.399; *p* = 0.035).

**Figure 5:**
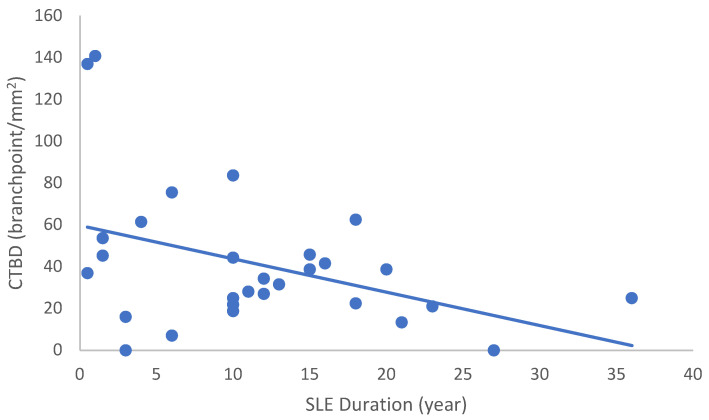
Correlation of CTBD with disease duration (r = −0.415; *p* = 0.028).

**Figure 6:**
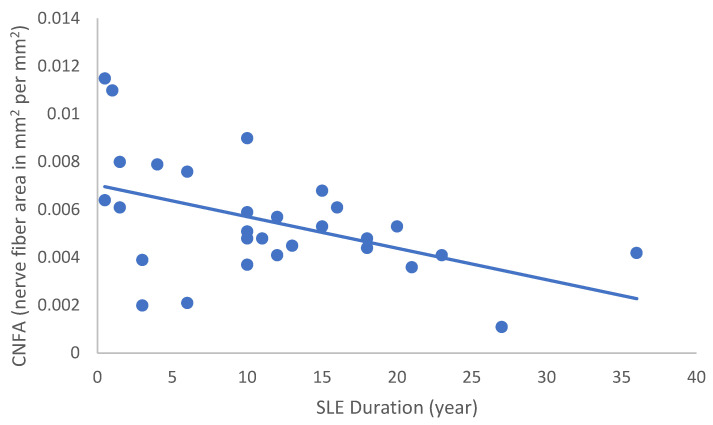
Correlation of CNFA with disease duration (r = −0.480; *p* = 0.010).

In the SLE group, none of the DED and confocal microscopic parameters had a significant correlation with age, SLEDAI score, or DED parameters. CNFD had a negative correlation with the central LCD (r = −0.37, *p* = 0.048) (Figure 2). Furthermore, CNBD (r = −0.389, *p* = 0.040) (Figure 3), CNFL (r = −0.399, *p* = 0.035) (Figure 4), CTBD (r = −0.415, *p* = 0.028) (Figure 5), and CNFA (r = −0.415, *p* = 0.028) (Figure 6) had a negative correlation with disease duration. To further interpret the results, it is important to highlight that we did not use any corrections regarding the subsequent statistical correlation calculations to minimize Type 1 statistical error. However, if the corrected *p* values, according to Bonferroni correction *p* < 0.0125, would be used for the correlation, then the results should be interpreted accordingly.

## 3. Discussion

The cornea is the most innervated tissue in the human body, with a nerve density of 300 to 600 times that of the skin [22]. Around seventy branches of the long and short ciliary nerves enter the peripheral corneal stroma, forming the sub-basal nerve plexus between Bowman’s layer and the basal epithelium [22]. Intact corneal innervation is of paramount importance for the maintenance of the corneal structure, providing protective and trophic functions to epithelial integrity as well as inhibiting exaggerated immune reactions secondary to exo- or endogenous triggering mechanisms via parasympathetic/sympathetic neurotransmitters [23,24].

Until recently, the cornea was viewed as an immune-privileged site of the human body mainly due to the fact that neither lymphatic nor blood vessels were detected in the unharmed tissue. However, Hamrah et al. showed that the cornea contains immature resident immune cells that do not express major histocompatibility complex class II in a latent, inactive ”state”. These DCs mature after inflammation or transplantation and subsequently move into draining lymph nodes [25]. Of note, Piezo1 channels are crucial mechanosensory players in lymphatic mechanotransduction [26].

DCs are potent antigen-presenting cells that can mediate both innate and adaptive immune responses by stimulating T cells. Given their strategic location in the corneal epithelium and anterior stroma, they may be the central player in orchestrating the ever-complex immunological interactions on the ocular surface. Recently, in vivo confocal microscopy (IVCM) has evolved into the best noninvasive in vivo imaging technique on hand that can provide us with a resolution of images comparable to that using ex vivo histochemical methods. IVCM allows the systematic study of corneal cellular components and enables the quantification of nerve morphology and density, as well as the study of immune cells such as corneal epithelial DCs in human subjects [27]. Investigation of corneal DC and nerves using IVCM has clinical relevance in various ailments [28,29]. Despite the clinical strength and abilities of IVCM to depict minute changes in cellular interactions, there have been few reports using IVCM to study the correlation between the immune and nervous systems in the human cornea.

There are different types of corneal nerves innervating the cornea. The majority of them are polymodal nociceptors (around 70%) that can express a wide variety of transducing ion channels in their nerve terminals, such as TRPA1, TRPV1, ASIC, and Piezo2. They can be activated not only by mechanical forces and heat, but various exogenous and endogenous chemicals [30]. Mechanonociceptors, expressing Piezo2 channels, can make up 15% of corneal nerve fibers [17,31], rendering them to be crucial players in maintaining corneal homeostasis. Mechanonociceptors can respond to sustained mechanical stimulus via A-delta fibers signaling the presence and velocity of change in the mechanical force [32,33]. These relatively rapidly adapting mechanosensitive fibers contribute to the pain experienced when a foreign body touches the ocular surface.

A higher density of DCs in the corneal center and periphery has been brought into association with many immune-related diseases, including RA [34], SLE [35], ankylosing spondylitis [36], and type 1 diabetes. Ferdousi et al. indeed demonstrated lower CNFD with a higher density of both immature and mature DCs in the cornea of type 1 diabetic children and adolescents [37]. We found a lower density of corneal nerves in the corneal center, along with a negative correlation between disease duration and four out of five confocal nerve parameters that are in line with their and Bitirgen’s findings [38]. Another study by Bitirgen et al. suggested a relationship between immune cells and small nerve fiber integrity in SLE patients [39]. We know from previous studies that there is a significant subclinical small fiber neuropathy in SLE patients [40,41]. We found no difference in total and immature LC densities between patients with SLE and control subjects in our study that might be attributable to the significant number of patients receiving immunosuppressive medications.

There is growing evidence now that DED is not only connected with a higher density of immune cells in the cornea, but that there is a marked reduction in many corneal nerve-related parameters that may contribute to the diseased state of the ocular surface [42]. The exact relationship between corneal nerve fiber length, corneal nerve density, and corneal DCs is yet to be fully elucidated; however, it seems quite reasonable to regard them as one of the most important players in shaping the ocular surface’s homeostasis through the Piezo system, as was postulated earlier [15]. Hence, the disruption and disturbance of this delicate crosstalk between Piezo2 and Piezo1, and the resultant impaired crosstalk between corneal nerves and immune cells, may result in the development of various DED-related symptoms, including gritty sensation, irritation, redness of the eye, etc. Indeed, corneal nerve damage can rob the eye of its immune privilege through the generation of antigen nonspecific CD11c+ contrasuppressor (CS) cells that can downregulate CD103 on Tregs, subsequently preventing the induction of anterior chamber-associated immune deviation (ACAID) [43].

In this study, we used OSDI to assess the symptoms of ocular irritation and the effects it has on the patient’s vision. We found higher OSDI scores in the SLE group; however, we failed to find a correlation between nerve density and dry eye-related symptoms. Tepelus et al. found significant negative correlations between corneal nerve density and DC density and between corneal nerve density and OSDI scores in patients with non-Sjögren’s (NSDE) and Sjögren’s syndrome dry eyes (SSDE) [29]. Labbé et al. found a correlation between corneal nerve length and OSDI, but failed to show a correlation between corneal nerve density and OSDI score in patients with NSDE [44]. Others found that corneal nerve length was negatively correlated with sensitivity to light, and nerve width was positively correlated with the OSDI score, painful eyes, and blurred vision [45]. We must point out that these above-mentioned studies did not explore the DC status of the cornea, but solely targeted the corneal nerves as an area of interest, along with giving a comprehensive description of the patient’s symptoms. We believe that the neuro-immune crosstalk is important to learn more about these intimate homeostatic mechanisms operating on the ocular surface. We further investigated our SLE group creating two subgroups according to the disease activity. We failed to find a significant difference either in the corneal nerve parameters or in the LC parameters, irrespective of their clinical status. This can be explained by the complex, ever-changing nature and interactions of the cellular participants of the cornea. Indeed, Liu et al. showed that the correlation between DC number and corneal nerve density can be time and exposure dependent in contact lens wearers [46]. This highlights the importance of corneal nociceptors and mechanoreceptors in the cascade of events influencing the immune status of the cornea, starting from the suggested disruption of Piezo2–Piezo1 crosstalk and leading to the resultant dysregulation and imbalance in the immune–neuro crosstalk. The chronic pain found in SLE patients might be related to clinical and subclinical small fiber neuropathy, and neurotrophic pain can develop as a longstanding consequence. Indeed, there is strong evidence that the peripheral nervous system regulates innate immune reactions, and dysfunction of the peripheral nervous system may result in proinflammatory immunological responses, termed “neurogenic inflammation” [47].

However, it is also important to highlight where this autoimmune pathophysiology could start; hence, what could be the primary damage that initiates the disease process. Peripheral neuropathy in SLE shows a rather complex view and hardly ever shows the picture of pure sensory neuropathy [48]. Of note, in general, the sensory nerves of autoimmune diseases are affected in an analogous complex way, but the proprioceptive and vibration senses are impaired the most [49]. Even more interesting is that the principal mechanotransduction ion channel responsible for proprioception is Piezo2 [11], not to mention the function of Piezo2 in vibration detection [50]. Recently it was theorized that the autologous channelopathy of Piezo2 at corneal somatosensory terminals contributing to proprioception could be the primary damage of a quad-phasic non-contact injury mechanism in DED [16] and in autoimmune conditions [12,13]. The primary microdamage of these type of channels are suggested to be their over-excessive mechanotransduction under allostatic stress, leading to a pathophysiological overreaching response [51]. Of note, in general, Piezo channels can be activated by stretch, shear stress, and indentation [52,53,54]. Moreover, this type of painless proprioceptive terminal Piezo2 primary damage is proposed to be one principal gateway to pathophysiology, and the gateway to remodeling and regeneration as well [19]. Not to mention that Piezo2 channelopathy is also suggested to be associated with impaired crosstalking between Piezo1 and Piezo2 [15], beyond being a principal transcription activator [19].

The primary damage is a painless transient one; however, underlying genetic risk factors and environmental triggers, such as repetitive non-contact re-injury, could lead to chronification as the tertiary phase of these microinjuries [16]. Correspondingly, genetic inheritance and environmental risk factors play a critical role in this chronification process in SLE [1]. Furthermore, Toll-like receptors (TLR) [15] and damage-associated molecular patterns (DAMPS) [16] play an underlying pathogenic role in the evolvement of this tertiary injury phase [16]. High-mobility group box 1 (HMGB1), a nuclear protein involved in transcription regulation, is a good example of DAMPS, since its expression is increased not only in SLE [55], but in DED as well [56]. Of note is that HMGB1 may already have a role in the secondary phase of this quad-phasic non-contact injury phase, since it is reported that HMGB1 level shows a correlation with delayed onset muscle soreness (DOMS) hours after exercise [57]. It is also known that increased HMGB1 could result in elevated cytokine release via TLR-4 signaling [58]. Even more importantly, the increased HMGB1 level shows a correlation with disease activity in SLE [59]. Moreover, animal research showed that increased TLR-4 contributes to the inflammation of DED [60]. Overall, an activated Hsp70/TLR4/Interleukin-6/TNF-α signaling pathway is emerging as a result of the primary damage [14], and this activated pathway certainly contributes critically to the evolvement of SLE. Indeed, the polymorphism of the Hsp70 gene locus shows a strong genetic link with SLE [61] and serum HMGB1 level shows a high correlation with TNF-α, representing the disease activity in SLE [59].

Another consequence of the acute Piezo2 channelopathy at the proprioceptive sensory terminals is the increased level of natural killer T (NKT) cells [14]. These cells are the first line of defense of the innate immune system, and they respond well to microbial-presented pathogen-associated molecular patterns. NKT cells are even shown to have anti-inflammatory nature in diseases, like in SLE [62] and in RA [63,64]. However, these cells are implicated in going through depletion throughout the chronification of Piezo2 microinjury [13]. Accordingly, the level of NKT cells is decreased in SLE [62,64,65,66]. Of note is that one subtype, called iNKT cells, have the potentials to activate not only T lymphocytes, but B lymphocytes as well [64], therefore further constructing the link between the innate and adaptive immune system [67] at the very early stage of the disease process. Accordingly, research showed that not only did iNKT cells decrease substantially in SLE [62,65,66], but their shortage correlated with SLE disease activity [62,64]. This drop of NKT cells in SLE is also associated with increased levels of IgG [68]; hence, they could even contribute to autoantibody production. Moreover, it has been demonstrated that PIP_2_ and PIP_3_ equilibrium regulates B lymphocyte activation [69], as PIP_2_ also plays an important role in Piezo2-dependent mechanotransduction [70]. Hence, the current authors propose that antiphospholipid antibodies could further imbalance the PIP_2_ and PIP_3_ equilibrium in SLE, therefore not only contributing to Piezo2 channelopathy chronification, but also to additional activation of B lymphocytes.

The iNKT cell reduction in SLE also contributes to Th1/Th2 imbalance [64]. Furthermore, iNKT cells are also shown to facilitate the proliferation and maturation of DCs, and the initial increase in these cells could play a role in Th1/Th2 imbalance as well [71]. Of note, this could be another underlying reason behind our earlier observations that a significant number of LCs migrate to the central cornea as a maturation process in DED of SLE [35]. In addition, certain iNKT cells not only facilitate autoantibody production, but also secrete IL-17 in SLE when gene susceptibility is present [72], and this production is shown to be proinflammatory dependent beyond being intrinsic [73].

Autoantibodies production in SLE has dominantly taken presence in the form of antinuclear antibodies in response to antigen-presenting cells, and the excessive autoantibody production is likely to be the result of the insufficient self-eating of cellular apoptotic degradational products [1]. Hence, the aetiopathogenesis of SLE could be considered an autoimmune disease where the clearance of apoptotic degradational materials is captivated by antigen-presenting cells instead of by proper phagocytic self-eating [1]. This incomplete self-eating process has been postulated in another autoimmune disease recently with a critical neurocentric relevance [13]. Of note, neuron terminal Piezo2 channelopathy contributing to proprioception is suggested to be a principal transcription activator leading to access to underlying genetic variants and cell type-specific noncoding DNA mutations to be expressed [19]. Moreover, the same Piezo2 channelopathy also disrupts the Piezo2–Piezo1 crosstalk; therefore, if the underlying genetic variants and cell type-specific noncoding DNA mutations impede proper regeneration, then the wound healing process is kept alive [16]. In support of this theory, there are indeed three main SLE susceptibility loci, namely the TLR/type I IFN pathway, immune-complex processing, and immune signal transduction [74].

Important to note that autoantibodies are also produced against other targets in SLE. Close to 20% of SLE patients show elevated levels of antiphospholipid antibodies, hence linking the disease to antiphospholipid syndrome [75] and upstream to supraspinal neural lupus [76,77]. As aforementioned, antiphospholipid antibodies could contribute indirectly to chronic Piezo2 channelopathy and B lymphocyte activation. Moreover, autoantibodies against vascular heparan sulfate proteoglycans (HSPG) at the vascular basement membrane [78] could reveal another important aspect of the pathophysiology of SLE since proteoglycans are proposed to be an essential medium of the extracellular matrix in the re-establishment of proper Piezo2–Piezo1 crosstalk and regeneration [16]. In favor of this theory, HSPGs are indeed critical mediators of stem cell function [79], as do Piezo1 as well [80]. Furthermore, autoantibodies attack glutamate receptors, like NMDA receptors, in SLE, leading to damage to the central nervous system and to cognitive impairment [81,82], not to mention their pathomechanistic role in the flare-ups in SLE [83]. Of note is that NMDA receptor activation is suggested to be an important consequence of somatosensory terminal Piezo2 channelopathy and DED [16].

The altered expression and dysfunction of the K_2P_ potassium ion channel family may also play a critical role in the pathophysiology of autoimmune diseases [84], and it is known that both Piezo1 and Piezo2 channels enhance the mechanogating of K_2P_ channels [85]. Recently, we emphasized the relevance of Piezo2 channelopathy-induced activation of the K_2P_-TASK1 signaling axis in the pathophysiology of RA [15]. Interesting to note that TASK1 and K_v_1.3 compensate TASK2 imbalance [86]. Moreover, elevated expression of K_v_1.3 has long been associated with pathogenic autoreactive T cells, like in multiple sclerosis and RA [87,88], and the inhibition of K_v_1.3 reduces neuroinflammation by rectifying calcium signaling alteration [89]. During B cell differentiation, only a subset, namely quiescent class-switched memory B cells, maintains high K_v_1.3 expression during memory B cell activation [90], constructing an active neuroimmune axis between the central nervous system and periphery in autoimmune diseases [91]. Even more importantly, the activation or expression of K_v_1.3 channels on T cells of SLE are not different from normal resting T cells; however, SLE alters the kinetic dynamics of K_v_1.3 channel compartmentalization in the immunological synapse [92]. This may have additional relevance on T cell function regulation, which not only could contribute to the dysregulated balance of Th17 and Th1 cells and increased Th17 cell response in SLE [93], but could also impact B cell differentiation and activation in the aforementioned pathway. Indeed, the genetic elimination of K_v_1.3 channels in T lymphocytes alleviated disease manifestation in SLE [94]. In summary, the suggested primary damage in DED, namely the corneal mechanosensory Piezo2 channelopathy, is independent of SLE and disrupts neural regeneration on the chronic path by impairing the Piezo2–Piezo1 crosstalk. As a result of this sustained chronification process, the current authors expect a Piezo1 downregulation on corneal Langerhans cells, which in turn leads to cell activation on Langerhans cells. Accordingly, disrupted neural regeneration and increased Langerhans cell activation are the key findings of the current study. Furthermore, the SLE associated lost autoimmune neuroinflammation compensation, lost phagocytic self-eating capacity, and lost transcription regulation, not to mention autoantibodies against vascular heparin sulfate proteoglycans and phospholipids could all contribute directly or indirectly to the observed progressive fashion of DED in SLE patients of the current study. It is important to note that this was a cross-sectional study conducted earlier with an aim to investigate the relationship between corneal DCs and dry eye parameters in SLE patients. Analysis of the confocal images from a neurocentric point of view was not part of the initial research design, and this may present an obvious drawback of our investigation. The findings could therefore be viewed as a preliminary confirmation of a theory, and more research is advised with a specially adapted research strategy and a greater emphasis on identifying the molecular background of this altered corneal neuro-immune status. Not to mention, there is a need for future in-depth investigation of the molecular upstream mechanisms to establish the consequence of impaired Piezo2–Piezo1 crosstalk, e.g., analyzing the expression level of Piezo1 and activation of Langerhans cells in established cell models.

## 4. Materials and Methods

### 4.1. Patients

Twenty-nine SLE patients and twenty-nine age- and gender-matched control subjects were enrolled in this cross-sectional, comparative study which was carried out in conformity with the most recent guidelines outlined in the Declaration of Helsinki. All participants provided their written informed consent, and this study was evaluated and approved by the Central Ethics Committee of Hungary (ETT TUKEB, 15410-2/2011-EKU). We used the American College of Rheumatology’s (ACR) 1997 modified criteria for SLE diagnosis and classification [95]. Individuals with a previous history of ophthalmic surgery, uveitis, corneal edema, haze, or previous keratitis of any kind, along with contact lens users, were excluded from this study. Patients with secondary Sjögren’s syndrome diagnosed using the 2002 American–European consensus criteria for Sjögren’s syndrome were also excluded from the study [96]. Patients with Schirmer test results of less than 5 mm/5 min were not automatically excluded from the study if no anti-Ro(SSA) or anti-La(SSB) antibodies were detected, or other functional tests and histopathological tests did not support the diagnosis of Sjögren’s syndrome.

The SLE patients had different disease activities and durations. The disease activity for SLE was determined by a rheumatologist on the day of ocular assessments using the Systemic Lupus Erythematosus Disease Activity Index (SLEDAI) [97,98,99]. Patients were judged to be in remission if their SLEDAI score was 0, and disease activity was considered to be a score higher than 0.

### 4.2. Dry Eye Examination

In our earlier works [34,36], we have provided in-depth descriptions of the protocols we followed in our studies to identify individuals with dry eye disease. In short, lid-parallel conjunctival folds (LIPCOF) were identified at the temporal aspect of the lower eyelid edge according to the procedure established by Pult et al. [100]. Without anesthesia, tear production was assessed using a Schirmer test strip (Haag-Streit UK Ltd, Bishop’s Stortford, UK, ref. 4701001). After inserting fluorescein dye drop into the lower conjunctival sac for one minute, the evaluation of tear break-up time (TBUT) was carried out. The three successive measurements’ mean value was identified as TBUT. With the help of the OSDI questionnaire, subjective discomfort was assessed [101].

Only the right eye was subjected to examinations, which were conducted in a consistent setting and the same room.

### 4.3. In Vivo Confocal Microscopy (IVCM)

In vivo confocal corneal microscopy was used to assess the SBNP and LCs’ morphology and density. All patients underwent corneal laser scanning procedure by the Heidelberg Retina Tomograph with Rostock Cornea Module (HRT III RCM) manufactured by Heidelberg Engineering Inc. in Heidelberg, Germany, incorporating Heidelberg Eye Explorer version 1.5.10.0. The corneal microscope used a 670 nm red wavelength diode laser source and had a video control for viewing and allowing external manual fine-tuning for better focus level in the axial direction, producing images of the cornea with exact information on the depth of the investigation. The main area of interest was at the level of the subbasal nerve plexus, around 50–60 µm from the corneal surface. The images from the laser confocal microscope depict a coronal slice of the cornea with a size of 400 µm × 400 µm at a selectable corneal depth, separated from adjacent images by around 1 to 4 m, and with a lateral resolution of 1 µm/pixel. Topical anesthetic eye drops (Oxybuprocaine–Humacain 0.4%, Human Pharmaceuticals, Gödöllő, Hungary) were used to numb the ocular surface.

### 4.4. Imaging Strategy

Due to its specific placement in the inferocentral cornea, the whorl of the sub-basal nerve plexus serves as a stable, dependable landmark during imaging. The fixation target of the IVCM was manually adjusted for each participant to line up with the central corneal reflex while taking images. For the periphery, we identified the corneal center first and then asked the patient to look at a target moveable red light with their contralateral eye to keep the fixation of that eye in place. To maintain a constant distance between the microscope head and the ocular surface, a disposable plastic cap (TomoCap; Heidelberg) was utilized. As a coupling medium, one drop of artificial tear gel (0.2% carbomer, Vidisic^®^, Chem.-pharm. Fabrik GmbH, Brunsbütteler, Berlin, Germany, Bausch&Lomb) was used to ensure airless contact. Two-dimensional en-face images were taken at the corneal center and at 6 o’clock limbal area using a 400 µm field of view lens. Every examination was conducted in the same room with the same setup.

### 4.5. Imaging Analysis—SBNP Analysis

ACCMetrics software was utilized to evaluate the morphometric parameters of the SBNP [53]. A set of images (between 5 and 10) best representing the SBNP were collected. For the SBNP analysis, the following parameters were automatically quantified by the software: CNFD; CNFL; CTBD, and corneal nerve fiber width (CNFW, the average nerve fiber width mm/mm^2^) for each image, and the data of the images were averaged.

### 4.6. Imaging Analysis—LC Analysis

Morphological characteristics of “LC activation” shown on IVCM (size, number, and length of dendrites) were proved to mirror the immunological status of the cornea and seem to be comparable to those found during immunohistochemistry investigations. Immature or inactive DCs (DC-SIGN+, HLA-DR−) have brighter cell bodies and none or shorter dendrites on IVCM. Mature or active DCs (DC-SIGN+, HLA-DR+) have longer interdigitating dendrites and are more predominant in individuals with inflamed corneas [101].

According to the protocol developed by Zhivov et al. [102,103], LC densities were assessed in the corneal center and at 6 o’clock corneal periphery. Thirty photos of the right eye were collected, and the five best-focused images were used for the analysis in the previously reported masked manner (an impartial evaluator was shielded from the patient’s medical history or ophthalmological state) [34,36]. After choosing the area of interest and identifying LCs (bright, mainly oval or elongated particles with a diameter of up to 15 m), cells were manually noted, and the program automatically determined cell density (cell number/mm^2^). On a 0–3 scale, LCs morphology (LCM) was assessed. Using the built-in caliper, the length of every dendrite from every LC was measured in each image. The absence of LCs in the cornea was indicated by a score of 0. Whenever cells lacked processes, a score of 1 was assigned. If the length of the processes did not reach the largest diameter of the cell body, a score of 2 (small processes) was awarded. If the processes were longer than the maximum diameter of the cell body, a score of 3 (long processes) was calculated. The maturation of the LCs at both regions of the cornea was described using the average of LCM, which was determined for each of the figures chosen.

### 4.7. Statistical Tests

Software STATISTICA version 11.0 (StatSoft, Tulsa, OK, USA) was used to conduct the statistical analysis. Using the Mann–Whitney U test, the control and SLE groups were compared. The Wilcoxon test was used to compare the values for LC density and morphology in the central and periphery. We used the Kruskal–Wallis and Mann–Whitney tests for subgroup analysis. In order to compare groups based on the presence or absence of LC in the cornea, the Fisher exact test was used. *p* 0.05 was regarded as significant in each test.

## Figures and Tables

**Table 1:** Demographic and clinical data in the control and SLE groups. Data are shown in mean ± SD values; SLE disease activity index (SLEDAI); tear break-up time (TBUT); lid parallel conjunctival folds (LIPCOF); ocular-surface disease index (OSDI); and NA means not applicable. * *p* < 0.05 Mann–Whitney test.

	Control	SLE	*p*
No. of patients	29	29	NA
No. of eyes	29	29	NA
Age (years)	44.5 ± 19.8	45.2 ± 11.8	0.907
Gender (male/female)	11/18	1/28	NA
SLE duration (years)	-	11.5 ± 8.6	NA
SLEDAI	-	2.6 ± 0.9	NA
TBUT (s)	11.5 ± 3.0	6.9 ± 3.7	0.001 *
Schirmer test (mm/5 min)	12.2 ± 3.1	8.6 ± 10.11	0.006 *
LIPCOF	1.0 ± 0.6	1.6 ± 0.7	0.004 *
OSDI	9.1 ± 6.5	29.3 ± 21.2	0.001 *

**Table 3:** Mean and SD values for the demographic and clinical data, and confocal microscopic findings of the SLE subgroups, with *p* values of the comparisons (Mann–Whitney test). CNFD: the number of fibers per mm^2^; CNBD: the number of branch points on the main fibers per mm^2^; CNFL: the total length of nerves in mm per mm^2^; CTBD: the number of branch points per mm^2^; and CNFA: the total nerve fiber area in mm^2^ per mm^2^. LCD: Langerhans cell density. LC Morph: LC morphology. NA: not applicable. Mann–Whitney test.

	SLEDAI = 0	SLEDAI > 0	*p*
No. of eyes	7	22	NA
Age (years)	51.0 ± 7.6	43.3 ± 12.3	0.176
SLEDAI	0	1.0 ± 0.7	NA
SLE duration (years)	13.8 ± 4.6	10.8 ± 9.5	0.229
TBUT (s)	4.8 ± 1.8	7.5 ± 3.9	0.130
Schirmer test (mm/5 min)	6.6 ± 7.2	9.3 ± 10.9	0.471
LIPCOF	1.8 ± 0.7	1.5 ± 0.7	0.144
OSDI	27.7 ± 25.9	29.7 ± 20.1	0.740
CNFD	17.4 ± 5.6	14.6 ± 7.2	0.346
CNBD	23.8 ± 13.7	21.9 ± 18.5	0.575
CNFL	12.1 ± 23.6	29.7 ± 20.1	0.610
CTBD	39.2 ± 21.4	42.0 ± 37.2	0.779
CNFA	0.005 ± 0.002	0.006 ± 0.003	0.939
Central LCD	32.7 ± 28.9	33.0 ± 35.9	0.610
Peripheral LCD	95.0 ± 40.4	91.2 ± 61.6	0.600
Central LCM	1.0 ± 0.0	1.1 ± 0.6	0.610
Peripheral LCM	1.4 ± 0.5	1.4 ± 0.7	0.593

## Data Availability

The data presented in this study are available on request from the corresponding author.

## Abbreviations

CNBD	corneal nerve branch density
CNFA	corneal nerve fiber area
CNFD	corneal nerve fiber density
CNFL	corneal nerve fiber length
CTBD	corneal nerve fiber total branch density
DAMPS	damage-associated molecular patterns
DC	dendritic cell
DED	dry eye disease
DOMS	delayed onset muscle soreness
HMGB1	high-mobility group box 1
HSPG	heparan sulfate proteoglycan
IVCM	in vivo confocal microscopy
LC	Langerhans cells
LCD	Langerhans cell density
LCM	Langerhans cell morphology
LIPCOF	lid-parallel conjunctival folds
NKT cell	natural killer T cell
NSDE	non-Sjögren’s dry eye
OSDI	ocular-surface disease index
PACAP	adenylate cyclase-activating polypeptide
RA	rheumatoid arthritis
SLE	systemic lupus erythematosus
SLEDAI	Systemic Lupus Erythematosus Disease Activity Index
SSDE	Sjögren’s syndrome dry eyes
TBUT	tear break-up time
TLR	Toll-like receptor
VIP	vasoactive intestinal peptide
